# Catalepsy or Death

**Published:** 1889-05-25

**Authors:** 


### The Hospital

A Weekly Institutional Journal of

Science, Medicine, Nursing, and Philanthropy.

*For Hospitals, Asylums, Medical Practitioners, Students, Nurses, Families, Parishes, Congregations, and the Charitable Public.*

Vol. VI. No. 139.] [May 25, 1889.

### Catalepsy or Death.

A painful sensation has been experienced by the newspaper reading world in the reported possibility that Mr. Irving Bishop, the well-known thought-reader, was dissected by certain New York physicians whilst in a state of catalepsy, and not actually dead. The alarming thought at once forces itself upon every reader, "Is it possible that high authorities in the medical world may be deceived, and that a living man may be mistaken for dead, then dissected, and so killed ?" The bare possibility is enough to overwhelm sensitive minds with horror. The question is really not one but two; and must be considered in both its aspects. Is it possible, we ask first, that men of admitted status in medicine could be deceived as to the actual fact whether a man was dead or not ? And secondly, is it conceivable that medical men of that class could be in such haste to make a dissection of any particular human body as not to take sufficient care to assure themselves absolutely that death had occurred ? To both of these enquiries we emphatically answer "No, it is not conceivable," and we await the full account of this case with a confident hope that the New York physicians will be exonerated from both suspicions.

But whilst scientific medical experts will feel that no such deed of horror as the dissection of a living man by too eager physicians has actually been done, yet the history of strange and sudden deaths in general, and of Mr. Irving Bishop's life peculiarities and death in particular, are quite sufficient to warrant in his bereaved wife's mind the haunting thought that perhaps life was not extinct, and that the fate her husband most dreaded throughout his life had finally come upon him. The public, however, seem hardly to realise the extreme sense of responsibility felt by all worthy medical men on occasions of dangerous illness, and at the time of death. Long before the actual moment of dissolution the conscientious physician notes with the most painful anxiety every symptom of his patient which threatens death, and every indication, however slight, which gives even momentary hope of recovery. The professional education of the humblest qualified practitioner has at different times of his student career taken him repeatedly over the ground which includes the modes and marks of death. One part of his curriculum has been devoted to medical jurisprudence, and that subject deals with every conceivable cause and manner of death, and with all possible circumstances that have any bearing thereupon. It is difficult even to imagine any other method of ascertaining without doubt or question that death has actually taken place than those already familiar to physicians, and con stantly put in practice by them.

Not only is this the case as regards medical education in general, but particular physicians from time to time take up the consideration of the question afresh, and carefully collecting all facts new and old which bear upon it, bring them by speech or pen before the whole profession, and thus ensure a rediscussion of the subject. Within the last few months so eminent and trusted a scientist as Dr Richardson brought the matter specially before the Medical Society of London in a highly practical paper, and elicited a discussion of great value, which was afterwards published. Dr. Richardson gave deeply interesting and instructive particulars as to certain circumstances associated with dead persons, which demand more than ordinary skill and judgment on the part of the certifying practitioner. Some of these it will be well for non-professional, as well as professional readers to consider and bear in mind.

It will sometimes happen, for example, that a person, though really dead, will change colour in the face or elsewhere, and the portion of the body so affected, instead of presenting the pallid hue of death, may be bright red. Dr. Richardson relates the case of a young woman in Dean-street, Soho, who had died of what was supposed to be suppressed scarlet fever. At the moment of death the face of the young woman was left very dark, but in the course of a few hours it became so life-like in colour and appearance that her friends not unnaturally thought she was coming back to life again. The mother of the dead girl especially would not be convinced that life was not returning; and even when post-mortem changes made it absolutely certain that her daughter was dead, she still held to the belief that there had been a temporary reanimation.

It is well understood, even by the least experienced person, that after death the body more or less quickly becomes quite cold, and this coldness is one of the most convincing evidences that death has really taken place. But cases are by no means infrequent which diverge from this rule, and the bodies of dead persons, for various reasons, may remain warm for a very long time. Dr. Richardson relates that he was once called to see a gentleman who had been seized with apoplexy, and had immediately fallen dead. But although death was undoubted, as the after events decisively proved, yet for a long time the dead man retained all the natural warmth of life. The explanation of this phenomenon is quite simple. As a rule, death which is absolutely sudden results from the abrupt arrest of the circulation in the cerebral centres. Such an arrest, though it kill the patient, is at first followed by a marked rise of temperature, due to vascular resistance; and this is maintained for so long a time that unscientific spectators may easily be deceived, and find it impossible to believe that the patient is really dead.

Many other deviations from the normal rule are familiar to persons who are accustomed to the chamber of sickness and death. Sudden spasmodic movements of face or limbs are by no means uncommon; and if these take place, as they often do, during alarming epidemics, when the dead are coffined and removed with unusual haste, it is easy to understand how many stories of persons buried, but not dead, have arisen and found ready credence in the excited public mind. Happily, these horrifying phenomena are to a large extent associated with and confined to one kind of death—viz., that from Asiatic cholera. That terrible scourge we are happily not very familiar with in this country, and as sanitation progresses towards a more perfect standard, there is every reason to hope that, at any rate in its epidemic form, it will ultimately cease to be known among us.

The only other form of simulated death we need notice here is the one which has given rise to the present alarm—catalepsy. The cataleptic state may be idiopathic or traumatic—that is, either natural to the person or due to a wound. Cases of idiopathic catalepsy are exceedingly rare. There is only one sign which may be relied upon to indicate with certainty that the cataleptic is still alive, and that is the continued persistence of normal animal warmth. Dr. Richardson states that he has only seen one such case during his long professional career. In the first instance, the cataleptic lay long without movement or sign ot life, except that he continued warm. He finally quite recovered from the attack, and then stated that, although he could neither move nor speak, he was conscious, and could remember afterwards almost all that had occurred during the time. A second seizure, however, followed some time afterwards, and from that he never rallied. He was for a long time kept under careful observation, but no sign of returning life appeared, and gradually general decomposition set in.

The absolute signs of death are few, and as a rule are such that they carry conviction to the uninstructed as well as to the instructed mind. Has the patient suffered from a dangerous illness? has he gradually got worse? has he hovered between life and death for hours, or possibly for days? has he at length definitely ceased to breathe? and has the pulse, with the heart, stopped beating? does he gradually become stiff and cold? and in the course of a few days are the signs of decomposition evident to the senses? Then he is dead, and all the grief and all the love in the world will not bring him back again. The subject is painful and need not be dwelt upon. It is considered here solely with reference to the present alarm, and for the guidance and comfort of non-professional readers. As we said at the outset, it is not conceivable that there can have been any doubt as to the certain death of Mr. Irving Bishop before the American physicians laid a single curious hand upon his body; nor can we believe it possible that in any English-speaking country a cataleptic who is not really dead can be treated other than as a cataleptic, and preserved from all harm until nature shows convincingly that life and hope have fled.

